# Changes in physical activity and risk of fracture: a Korean nationwide population-based cohort study

**DOI:** 10.1038/s41598-020-73495-1

**Published:** 2020-10-01

**Authors:** Sangsoo Han, Hae-Dong Jang, Sungwoo Choi, Gi Deok Kim, Kyungdo Han, Hyunwoong Lim, Bongmo Koo, Kyung Dae Min, Jae-Young Hong

**Affiliations:** 1 Department of Emergency Meidcine, Soonchunhyang University Bucheon Hospital, 170 Jomaru-ro, Bucheon, 14584 Republic of Korea; 2 Department of Orthopaedic Surgery, Soonchunhyang University Bucheon Hospital, 170 Jomaru-ro, Bucheon, 14584 Republic of Korea; 3 Department of Biostatistics, College of Medicine, Catholic University, 296-12 Changgyeonggung-ro, Jongno-gu, Seoul, 03083 Republic of Korea; 4 Department of Industrial Management Engineering, Korea University, Seoul, 02841 Republic of Korea; 5 Department of Orthopedics, Korea University Hospital, Ansan, 123, Jeokgeum-ro, Danwon-gu, Ansan-si, Gyeonggi-do 15355 Republic of Korea

**Keywords:** Health care, Medical research, Risk factors

## Abstract

Physical activity (PA) is one of the most important modifiable factors associated with fracture risk. However, the association between interval changes in PA and the risk of fracture remains unknown. We investigated the risk of fracture development according to interval changes in PA in middle aged and older individuals. In this nationwide cohort study of adults aged ≥ 40 years, more than 4.9 million individuals without fractures within the last year who underwent two consecutive national health screenings in Korea from 2009 to 2012 were identified. The risk of fracture between 2013 and 2016 according to interval changes in regular PA was prospectively analyzed. Compared to individuals with a continuous lack of PA, those with a decrease in PA (0.41/1000 person-years (PY) decrease in incidence rate (IR); adjusted hazard ratio (aHR) 0.975; 95% confidence interval (CI) 0.964–0.987), increase in PA (1.8/1000 PY decrease in IR; aHR 0.948; 95% CI 0.937–0.959), and continuous PA (3.58/1000 PY decrease in IR; aHR 0.888; 95% CI 0.875–0.901) had a significantly reduced risk of fracture. Interval changes in regular PA were associated with risk of fracture. Individuals who engaged in continuous regular PA exhibited the maximum protective benefit against fracture.

## Introduction

Fractures are one of the most important public health priorities, especially in older individuals. The risk of fracture increases in individuals aged ≥ 50 years; moreover, fractures are associated with serious disorders and diseases, as well as increased mortality^1,2^. Indeed, more than half of patients with hip fractures are unable to return to their pre-fracture states, and the mortality rate rises to 33% within 1 year of fracture^3–5^. Therefore, fractures are an important contributor to socioeconomic loss.

Physical activity (PA) is one of the most important modifiable factors associated with fracture risk, along with alcohol intake and smoking^6^. Previous studies have demonstrated that exercise can help prevent fractures; however, the majority of these studies have included only elderly populations and none have assessed how changes in PA affect fracture prevention^7,8^. Thus far, there remains no clear consensus regarding the frequency and intensity of PA recommended to prevent fractures. One study found that the minimum amount of exercise that affected bone mineral density (BMD) was 2.1–2.5 sessions per week; however, it did not explore how changes in exercise habits affect fracture occurrence^9^.

Here, we investigated a group of middle-aged and older patients with demographic data, health survey information, medical tests, and medical claims from the Korean National Health Insurance Service (K-NHIS) in South Korea. The primary aim of this study was to identify the relationship between interval changes in PA and the risk of fracture. The secondary aim was to identify differences in fracture incidence according to PA in different sex, age, and previous fracture history subgroups.

## Materials and methods

### Data sources

This nationwide population-based study was conducted in South Korea using data obtained from the K-NHIS. Two data sets were used: the Korean National Health Examination (K-NHE) database and the K-NHIS claims database. The K-NHE database was used to select the participants and obtain information regarding confounding variables. The linked K-NHIS claims database for the same participants was then used to evaluate the occurrence of fractures. Insured Korean adults aged > 40 years and employees aged > 20 years undergo regular health checkups provided by the K-NHIS every 1–2 years. The K-NHE data obtained through these checkups provide information concerning anthropometric measurements, smoking and alcohol consumption status, and medical history through self-reported questionnaires and laboratory findings^10^. This database and the aforementioned nationwide medical records were combined and analyzed to construct a cohort to investigate health problems with NHIS research approval. The K-NHIS is a mandatory national health insurance system that covers approximately 97% of the South Korean population for all medical procedures, except cosmetic surgery and treatment for injuries sustained in road traffic accidents. The remaining 3% of the population is included in the Medical Aid program. Patients enrolled in the K-NHIS pay for 30% of their total medical expenses, and medical service providers submit claims for reimbursement from the K-NHIS for the remaining costs. Thus, medical information from almost all patients treated by a healthcare institution is prospectively integrated into the database. The K-NHIS claims database includes extensive health information from approximately 50,000,000 South Koreans. Indeed, it contains data from all clinics and hospitals regarding diagnoses and comorbidities (coded in accordance with the 10th revision of the International Statistical Classification of Diseases and Related Health Problems [ICD-10]), demographic characteristics, prescriptions, medical services (i.e., treatments and procedures), and costs for inpatients (i.e., those admitted to hospital) and outpatients (i.e., those who received ambulatory care). Since 2015, the K-NHIS has provided a dynamic, nationally representative, retrospective cohort database that includes information from nearly the entire South Korean population, and is open to all researchers whose study protocols are approved by the official review committee.

### Study design and participants

In this study, relevant information in the K-NHIS cohort database was collected to determine key variables used for adjustment and subgroup analyses^11^. Sociodemographic variables were obtained including sex, age, and perceived family economic status. Respondents were asked to self-record their height and weight; their body mass index (BMI, kg/m^2^) was calculated based on these data. All BMI and body weight data were measured by trained examiners. The study population was subdivided into two groups according to age: (1) middle-aged group, 40–64 years; and (2) elderly group, age ≥ 65 years. The study population was also divided based on income, with the low-income level defined as income below the 20th percentile. Current drinker was defined as individuals who drank > 30 g of alcohol per day. Individuals with diabetes were defined as either patients prescribed anti-diabetic drugs who had ICD-10 codes (E11–E14) or as patients with fasting glucose levels > 126 mg/dL, based on data from the K-NHE database. The presence of hypertension was defined as a systolic/diastolic blood pressure ≥ 140/90 mmHg from the K-NHE database, or the presence of ≥ 1 claim per year for a prescription of antihypertensive agents using ICD-10 codes (I10–I13, I15). The presence of dyslipidemia was defined as a total cholesterol level ≥ 240 mg/dL in the K-NHE database, or the presence of ≥ 1 claim per year for the prescription of antihyperlipidemic agents using ICD-10 codes (E78). Chronic kidney disease (CKD) was defined as an estimated glomerular filtration rate < 60 mL/min/1.73 m^2^. These definitions based on ICD codes have been validated in previous studies^12,13^. Blood pressure was measured using a blood pressure monitor after 5 min of rest, and all blood tests were performed after 8 h of overnight fasting.

### Study cohort establishment

**Figure 1:**
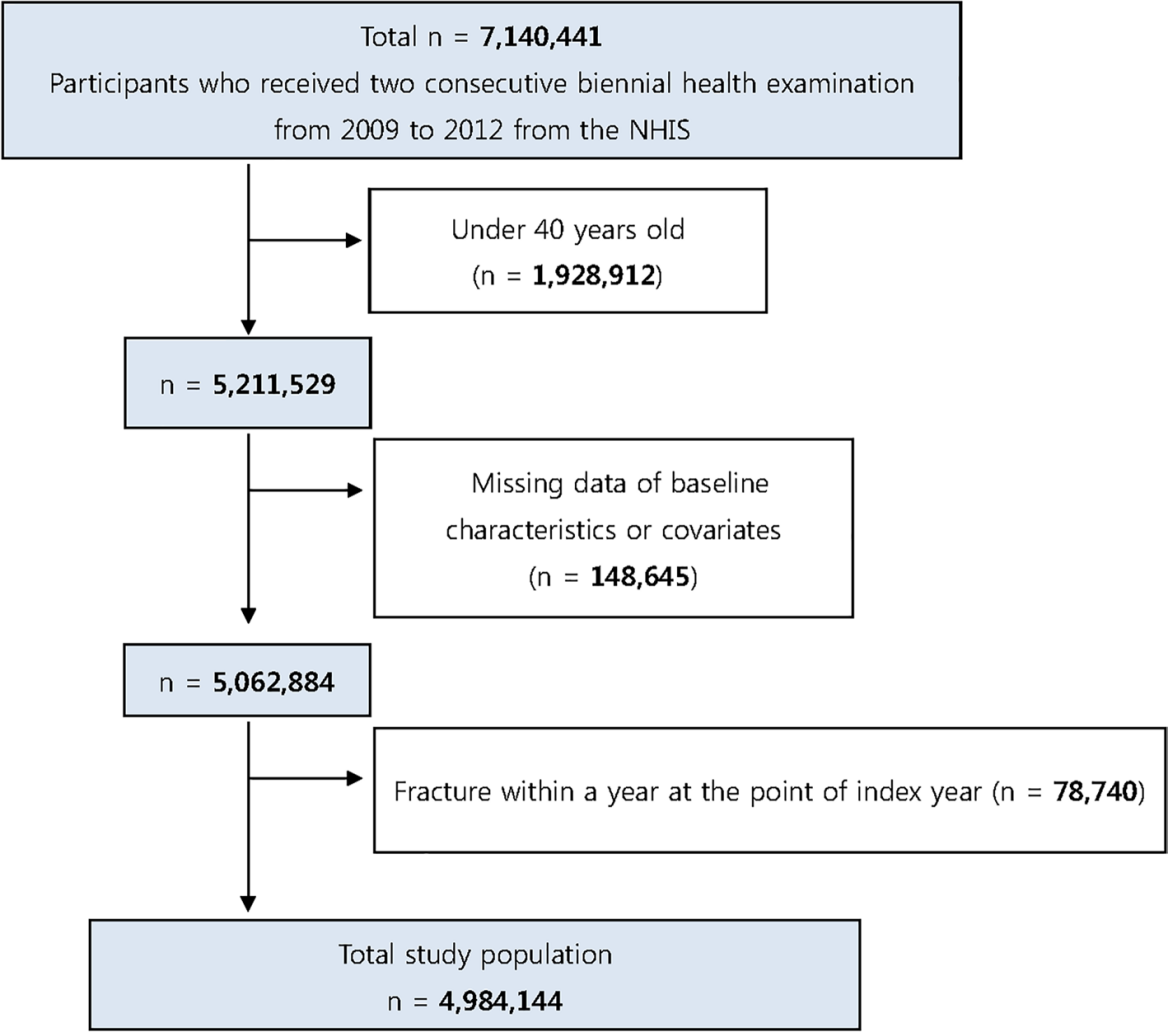
Flowchart of inclusion and exclusion criteria, based on the dataset of the Korean National Health Insurance Service (K-NHIS).

A total of 7,140,441 individuals who underwent two consecutive biennial K-NHEs from 2009 to 2010 and 2011 to 2012 were initially enrolled. Individuals aged < 40 years (n = 1,928,912) and those with any missing baseline characteristic data or health examination survey covariates (n = 148,645) were excluded. The linked K-NHIS claims database was then requested to determine the incidence of fracture in this cohort. To exclude pre-existing cases, individuals diagnosed with a fracture within 1 year of the index year were excluded (n = 78,740). A total of 4,984,144 participants were included in the final study population (Fig. 1).

### Classification of interval change in physical activity

During each K-NHE period, the participants in the K-NHIS cohort responded to a series of self-administered questionnaires regarding PA and other lifestyle behaviors. PA was measured using the Korean version of the International Physical Activity Questionnaire (IPAQ)-short version, which was designed for use in PA surveillance studies^13^. The IPAQ-short version consists of seven questions intended to determine the frequencies and durations of walking, moderate PA, and vigorous PA during the previous 7 days. The frequency of moderate PA was assessed with the following question: “In the past week, on how many days did you engage in 30 min or more of PA that increased your heart rate or breathing rate (e.g., brisk walking, playing doubles tennis, or cycling at a regular pace)?”. The frequency of vigorous PA was assessed with the following question: “In the past week, on how many days did you engage in 20 min or more of PA that was so vigorous it left you soaked with perspiration or breathless (e.g., running, aerobics, hiking, or fast cycling)?”. The response options ranged from 1 (none) to 6 (> 5 days per week).

Regular PA was defined using either of the two following criteria, based on the IPAQ Scoring Protocol: (1) vigorous intensity activity ≥ 3 days per week, or (b) moderate intensity activity ≥ 5 days per week^14^. The interval change in regular PA was evaluated among the participants at the two consecutive biennial K-NHEs. Categories were created as follows for change in regular PA at the second examination (2011–2012), compared to the first examination (2009–2010): (i) continuous lack of PA (regular PA (−) to (−)); (ii) decrease in PA (regular PA (+) to (−)); (iii) increase in PA (regular PA (−) to (+)); and (iv) continuous PA (regular PA (+) to (+))^15^.

### Follow-up for fracture outcomes

Information regarding medical claims records of the K-NHIS was collected to identify fracture events during the follow-up period, between January 1, 2013 and December 31, 2016. ICD-10 codes and hospitalization records from the K-NHIS system were used to identify the causes for total fracture events. Fractures were defined using ICD-10 codes, as follows: vertebral, S12.0, S12.1, S12.2, S22.0, S22.1, S32.0, M48.4, and M48.5; hip, S72.0, S72.1, and S72.2; and other fractures, including upper arm, S42.0, S42.2, and S42.3; forearm, S52.5 and S52.6; and lower leg, S82.3, S82.5, and S82.6. Individuals were deemed to have vertebral or other fractures when they had ≥ 2 outpatient visits within 12 months associated with the relevant ICD-10 codes. Hip fracture was defined as one hospitalization with a relevant diagnosis code. A fracture was defined as an instance of ≥ 1 of the above fracture types in an individual. If the participant died during the follow-up period, they were censored at the time of death. For the participants who experienced multiple incidences of fractures, first occurrence of fracture was considered as an event and follow-up was stopped.

### Statistical analysis

Baseline participant characteristics were calculated using numbers and percentages for categorical variables, and means and standard deviations for continuous variables. The characteristics of groups were compared using the chi-squared test for categorical variables and the t-test for continuous variables. Because the size of the total study population was large, nearly all variables showed significant differences according to the presence or absence of the outcome. The incidence rate (IR) was computed based on the prevalence of the outcome per 1,000 person-years (PY) from the number of total fracture events and the PY in each group, according to the change in PA status. Changes in IR were also assessed.

To evaluate the association between fracture risk and interval change in PA status between the two biennial K-NHE periods (2009–2010 and 2011–2012), Cox proportional hazards regression models were used; hazard ratios (HR) and 95% confidence intervals (CI) were calculated for each PA status, relative to the reference (continuous lack of PA). In Model 1, an unadjusted analysis was conducted by considering a range of covariates potentially associated with the development of fractures. In Model 2, an analysis was performed with adjustment for age and sex. In Model 3, further adjustments were made for lifestyle variables, including current smoking and alcohol consumption, as well as household income levels. In Model 4, a fully adjusted model was developed using BMI, dyslipidemia, and previous fracture within 3 years as the adjustment variables. To evaluate the robustness of the influence of clinical conditions on the association between interval change in PA status and fracture, the HRs for fracture in various subgroups were analyzed using Cox regression analyses with P-values for interaction. Subgroup analyses stratified by age (< 65 and ≥ 65 years), sex (male and female), BMI (< 25 and ≥ 25 kg/m2), household income level (income < 20th percentile or ≥ 20th percentile), cigarette smoking (current smoker or never smoker), current alcohol consumption (none or heavy), comorbidities (diabetes, hypertension, dyslipidemia, and CKD), and previous fracture (no or yes; within 3 years) were conducted. All statistical analyses were performed using SAS software (version 9.3; SAS Institute, Cary, NC, USA). Two-sided P-values < 0.05 were considered to indicate statistical significance for all analyses.

### Ethics statement

The study protocol was approved by the K-NHIS institutional review board. Anonymized and de-identified information was used for analyses; therefore, informed consent was not required. The study protocol was also approved by the Institutional Review Board of Korea University Hospital (Ansan, South Korea; approval no. 2020AS0030), and the study was conducted in accordance with the principles of the Declaration of Helsinki. All study procedures were performed in accordance with the relevant guidelines and regulations.

## Results

### Cohort characteristics

The study sample comprised 3,303,504 participants (66.28%) with a continuous lack of PA (regular PA (−) to (−)), 583,561 participants with a decrease in PA (regular PA (+) to (−)), 650,888 participants with an increase in PA (regular PA (−) to (+)), and 446,191 participants with continuous PA (regular PA (+) to (+)).

The demographic and clinical characteristics of the participants are compared in Table 1. The proportions of men, age subgroups, high BMI, and current smokers and drinkers, as well as mean age, BMI participants with low incomes, and participants with comorbidities (i.e., diabetes, hypertension, dyslipidemia, or CKD) were significantly different among the groups (P < 0.0001). Because the size of the total study population was large, all variables showed significant differences according to the interval changes in PA status. During the follow-up period, the prevalences of newly diagnosed fracture were 5.99% (n = 197,858) in the continuous lack of PA group, 5.84% (n = 34,066) in the decrease in PA group, 5.26% (n = 34,261) in the increase in PA group, and 4.52% (n = 20,188) in the continuous PA group. The incidence of fracture was highest in the continuous lack of PA group, which included participants who did not consistently exercise. Compared to the decrease in PA group, which included participants who had exercised continuously in the past but stopped, the incidence of fracture in the increase in PA group was significantly lower. The incidence of fracture was lowest (< 5%) in the continuous PA group, which included participants who had continued to exercise from the past to the present.

**Table 1:** Baseline characteristics of participants according to interval changes in physical activity status.

Interval changes in regular PA (from 2009–2010 to 2011–2012)	Continuous lack of PA (N = 3,303,504)	Decrease in PA (N = 583,561)	Increase in PA (N = 650,888)	Continuous PA (N = 446,191)	*P-value*
	PA (−) to (−)	PA (+) to (−)	PA (−) to (+)	PA (−) to (−)	
**Demographics**					
Male sex	1,687,642 (51.09)	322,733 (55.3)	360,244 (55.35)	288,410 (64.64)	** < 0.0001**
Age (years)	54.71 ± 10.53	55.95 ± 10.18	55.02 ± 9.91	55.14 ± 9.67	** < 0.0001**
**Age distribution**					
≥ 65 years	623,067 (18.86)	124,656 (21.36)	120,002 (18.44)	81,934 (18.36)	** < 0.0001**
BMI (kg/m^2^)	23.94 ± 3.05	24.16 ± 2.93	24.04 ± 2.88	24.16 ± 2.76	** < 0.0001**
High BMI (≥ 25 kg/m^2^)	1,120,785 (33.93)	212,040 (36.34)	223,493 (34.34)	159,046 (35.65)	** < 0.0001**
Current smoker	701,111 (21.22)	105,362 (18.06)	116,059 (17.83)	76,412 (17.13)	** < 0.0001**
Current drinker	1,391,493 (42.12)	250,991 (43.01)	293,956 (45.16)	231,673 (51.92)	** < 0.0001**
Low-income	2095 (29.84)	2095 (29.84)	8347 (23.78)	8347 (23.78)	** < 0.0001**
**Comorbidities**					
Diabetes	378,394 (11.45)	79,911 (13.69)	82,294 (12.64)	59,516 (13.34)	** < 0.0001**
Hypertension	1,094,622 (33.14)	212,878 (36.48)	224,188 (34.44)	158,971 (35.63)	** < 0.0001**
Dyslipidemia	820,600 (24.84)	156,172 (26.76)	166,169 (25.53)	114,208 (25.6)	** < 0.0001**
Chronic kidney disease	203,313 (6.15)	38,170 (6.54)	40,453 (6.22)	28,153 (6.31)	** < 0.0001**
**Fracture**					
Previous fracture (within 3 years)	99,987 (3.03)	17,532 (2.69)	17,376 (2.98)	10,148 (2.27)	** < 0.0001**
Incidence	197,858 (5.99)	34,066 (5.84)	34,261 (5.26)	20,188 (4.52)	** < 0.0001**

Means ± standard deviation.

Categorical variables: n (%).

Bold indicates a statistically significant result (P < 0.05).

*PA* physical activity; regular PA: high-intensity exercise ≥ 3 days per week for at least 30 min or moderate-intensity exercise ≥ 5 days per week for at least 20 min; *BMI* body mass index; current drinker: alcohol consumption > 30 g per day; low income: household income < 20% of median.

### Risk of fracture according to interval changes in PA status

Statistically adjusted Cox proportional hazards regression analyses were performed (Model 2, adjusted for age and sex; Model 3, adjusted for age, sex, smoking, alcohol consumption, and household income; and Model 4, adjusted for age, sex, smoking, alcohol consumption, household income, BMI, dyslipidemia, and previous fracture) to calculate HRs for newly diagnosed fractures in participants according to interval changes in PA (Table 2). The reference for fracture event HRs was participants with a continuous lack of PA. Participants with an increase in PA (regular PA (−) to (+)) and those with continuous PA (regular PA (+) to (+)) showed markedly lower risks of subsequent fracture development, despite adjustment for several potentially confounding variables (increase in PA group: adjusted HR 0.948; 95% CI 0.937–0.959; continuous PA group: adjusted HR 0.888, 95% CI 0.875–0.901) (Table 2). Therefore, the current study demonstrates a preventive effect of continuous PA or an increase in PA on fracture development.

**Table 2:** Calculated hazard ratios for fracture according to changes in physical activity status.

Changes in regular PA	Event (*n*) (fracture)	Total FU duration (PY)	IR (per 1000 PY)	Hazard ratio (95% CI)			
				Model 1	Model 2	Model 3	Model 4
Continuous lack of PA (PA (−) to (−))	197,858	14,014,911.3	14.12	1	1	1	1
Decrease in PA (PA (+) to (−))	34,066	2,483,882.1	13.71	0.971 (0.96–0.982)	0.965 (0.954–0.976)	0.971 (0.96–0.982)	0.975 (0.964–0.987)
Increase in PA (PA (−) to (+))	34,261	2,779,982.8	12.32	0.873 (0.864–0.884)	0.936 (0.925–0.947)	0.942 (0.931–0.953)	0.948 (0.937–0.959)
Continuous PA (PA (+) to (+))	20,188	1,914,666.5	10.54	0.748 (0.737–0.759)	0.867 (0.854–0.879)	0.877 (0.865–0.89)	0.888 (0.875–0.901)

Incidence rate = event (fracture) / total follow-up duration.

Model 1: Non-adjusted.

Model 2: Adjusted for age and sex.

Model 3: Adjusted for age, sex, smoking, alcohol consumption, and household income.

Model 4: Adjusted for age, sex, smoking, alcohol consumption, household income, body mass index, dyslipidemia, and previous fracture.

*PA* physical activity; regular PA: performing high-intensity exercise ≥ 3 days per week for at least 30 min or moderate-intensity exercise ≥ 5 days per week for at least 20 min; *FU* follow-up; *PY* person-year; *IR* incidence rate; *CI* confidence interval.

### Subgroup analyses for the risk of fracture according to interval changes in PA

**Figure 2:**
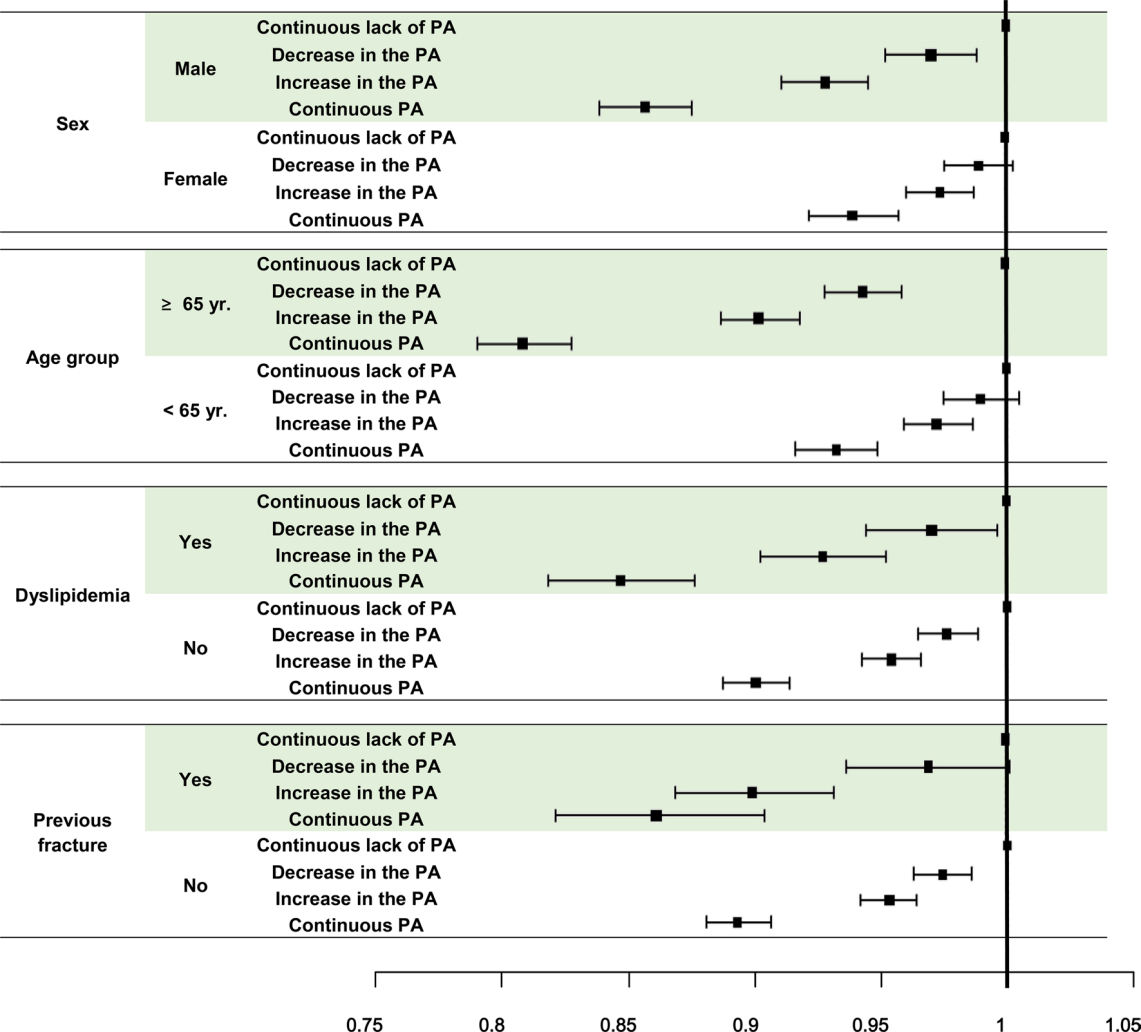
Fracture risk subgroup analyses according to changes in physical activity status.

Multivariable Cox proportional hazards regression analyses adjusted for confounding variables (age, sex, smoking, alcohol consumption, household income, BMI, diabetes, and hypertension) were used to estimate adjusted HRs for fracture, based on demographic characteristics and chronic comorbidities (Table 3 and Fig. 2). In subgroup analyses, the effect of continuous PA on fracture prevention was significantly higher among men (HR 0.85) than among women (HR 0.94) (P < 0.0001). Among the age subgroups, a significant prevention in the incidence of fracture was observed in the ≥ 65-year-old group (HR 0.80) compared to the < 65-year-old group (HR 0.93) (P < 0.0001). Moreover, subgroup analyses revealed significantly reduced HRs for the incidence of fracture in participants with previous fracture (HR 0.86) and dyslipidemia (HR 0.84), compared to participants without previous fracture (HR 0.89) or dyslipidemia (HR 0.90) (P = 0.0111 and P = 0.041) (Table 3). The increase in PA group and decrease in PA group also showed consistent trends. There were no significant differences in other subgroup analyses, which included BMI, smoking drinking, household income, diabetes, hypertension, and CKD (P > 0.05). In Fig. 2, the 95% CIs between categories overlap because the HRs for each category are represented by a single graph. Each subgroup category was analyzed independently.

**Table 3:** Subgroup analyses of risk for fracture according to changes in physical activity status.

	Changes in regular PA	n	Event (*n*) (Fracture)	Total FU duration (PY)	IR (per 1,000 PY)	Hazard ratio (95% CI)	*P-value*
**Sex**							
Male	Continuous lack of PA	1,687,642	60,572	7,200,983.0	8.41	1	< 0.0001
	Decrease in PA	322,733	12,366	1,380,043.0	8.96	0.97 (0.95–0.99)	
	Increase in PA	360,244	12,578	1,546,174.6	8.13	0.93 (0.91–0.94)	
	Continuous PA	288,410	9205	1,242,733.0	7.41	0.85 (0.83–0.87)	
Female	Continuous lack of PA	1,615,862	137,286	6,813,928.4	20.15	1	
	Decrease in PA	260,828	21,700	1,103,839.1	19.66	0.99 (0.98–1.00)	
	Increase in PA	290,644	21,683	1,233,808.2	17.57	0.97 (0.96–0.99)	
	Continuous PA	157,781	10,983	671,933.5	16.35	0.94 (0.92–0.96)	
**Age group**							
≥ 65 yr	Continuous lack of PA	623,067	94,244	2,516,088.0	37.46	1	< 0.0001
	Decrease in PA	124,656	15,327	512,563.4	29.90	0.94 (0.93–0.96)	
	Increase in PA	120,002	13,636	496,338.8	27.47	0.90 (0.90–0.92)	
	Continuous PA	81,934	7215	343,105.2	21.03	0.80 (0.78–0.82)	
< 65 yr	Continuous lack of PA	2,680,437	103,614	11,498,823.3	9.01	1	
	Decrease in PA	458,905	18,739	1,971,318.7	9.51	0.99 (0.97–1.01)	
	Increase in PA	530,886	20,625	2,283,644.0	9.03	0.97 (0.96–0.99)	
	Continuous PA	364,257	12,973	1,571,561.3	8.25	0.93 (0.91–0.95)	
**Dyslipidemia**							
Yes	Continuous lack of PA	378,394	31,662	1,574,149.7	20.11	1	0.041
	Decrease in PA	79,911	6065	334,950.7	18.11	0.97 (0.94–1.00)	
	Increase in PA	82,294	5641	346,689.0	16.27	0.92 (0.90–0.95)	
	Continuous PA	59,516	3317	252,542.1	13.13	0.84 (0.81–0.87)	
No	Continuous lack of PA	2,925,110	166,196	12,440,761.6	13.36	1	
	Decrease in PA	503,650	28,001	2,148,931.4	13.03	0.98 (0.96–0.99)	
	Increase in PA	568,594	28,620	2,433,293.8	11.76	095 (0.94–0.96)	
	Continuous PA	386,675	16,871	1,662,124.4	10.15	0.90 (0.88–0.91)	
**Previous fracture**							
Yes	Continuous lack of PA	99,987	23,284	379,208.7	61.40	1	0.0111
	Decrease in PA	17,376	3662	67,266.5	54.44	0.97 (0.93–1.00)	
	Increase in PA	17,532	3249	68,833.1	47.20	0.90 (0.86–0.93)	
	Continuous PA	10,148	1643	40,487.4	40.58	0.86 (0.81–0.90)	
No	Continuous lack of PA	3,203,517	174,574	13,635,702.6	12.80	1	
	Decrease in PA	566,185	30,404	2,416,615.6	12.58	0.97 (0.96–0.99)	
	Increase in PA	633,356	31,012	2,711,149.6	11.44	0.95 (0.94–0.96)	
	Continuous PA	436,043	18,545	1,874,179.1	9.90	0.89 (0.88–0.90)	

Incidence rate = event (fracture) / total follow-up duration.

Adjusted for age, sex, smoking, drinking alcohol, household income, body mass index, and diabetes mellitus.

*PA* physical activity; regular PA: performing high-intensity exercise ≥ 3 days per week for at least 30 min or moderate-intensity exercise ≥ 5 days per week for at least 20 min; *FU* follow-up; *PY* person-year; *IR* incidence rate; *CI* confidence interval; *yr.* years.

## Discussion

In this large population-based cohort study using the K-NHIS database, the relationship between interval changes in regular PA and fractures was assessed. Continuous regular PA, the key target, was associated with a significantly reduced risk of fracture in the general population. In contrast, the risk of fracture tended to increase in groups with reduced regular PA or a continuous lack of PA.

Our results regarding the association between reduced risk of fracture and regular PA are consistent with the findings of previous studies, in which high PA frequency was reportedly related to a lower risk of fracture. However, these studies only assessed PA at a single time point, rather than interval change in PA^7,9,16^. In our study, regular continuous PA was associated with reduced fracture incidence, compared with physical inactivity. This may explain why fracture incidence is often higher in older individuals, who typically have lower levels of PA than other members of the population^17^. In our study, continuous regular PA had a significantly greater effect on fracture prevention in men than in women, which may be due to larger increases in muscle mass (with similar exercise) in men than in women^18^. Muscles play important roles in protecting bones from severe trauma, stress, and strain, and the risk of fracture decreases with greater muscle mass^19^.

Continuous regular PA was associated with a greater reduction in fracture incidence in elderly individuals, defined as those > 65 years, compared with younger individuals. Indeed, fractures tend to occur more frequently in elderly individuals who have low BMD, and regular PA has been shown to increase BMD^17^. Therefore, regular PA may be effective for preventing fractures in elderly individuals. In particular, in one network meta-analysis, exercise was the only intervention that prevented fall-related injuries^20^. Moreover, a Cochrane meta-analysis found that exercise can reduce fractures associated with falls^21^. While it has been proposed that fractures occur in elderly individuals during PA due to a greater propensity for falls, various meta-analyses have reported that exercise has an anti-fracture effect in elderly individuals^20,22,23^. One meta-analysis suggested that participating in ≥ 3 sessions of PA per week increased the risk of falls in elderly individuals, while 2–3 sessions of exercise per week (each lasting 30–60 min) were safe and effective^24^. Although our study defined regular PA as high-intensity exercise ≥ 3 days per week for at least 30 min, or moderate-intensity exercise ≥ 5 days per week for at least 20 min, these amounts of PA may be excessive for elderly individuals.

The risk of fracture may increase by twofold in individuals with previous fracture, compared to individuals without previous fracture^25^. This effect is reportedly caused by low BMD, as well as changes in cancellous microarchitecture and damage to cortical bone (due to the fractures themselves or immobilization secondary to fracture)^26^. In addition, the risk of falls increases and the protective response against damage becomes impaired in individuals with previous fractures^27–30^. In the current study, individuals with previous fractures had higher anti-fracture effects, compared to individuals without previous fractures, in the continuous regular PA group. Therefore, patients with fractures should exercise regularly after acute treatment is completed. Notably, in our study, the anti-fracture effect of continuous regular PA was greater in the group with dyslipidemia than in the group without dyslipidemia. This is presumably because patients with dyslipidemia have lower BMD and higher incidence of fracture due to alterations in lipid metabolism^31^. Therefore, exercise is more important in patients with hyperlipidemia.

Several mechanisms may explain our findings regarding the association between interval changes in regular PA and fracture risk. First, PA has been shown to increase bone size and BMD^32,33^. Moreover, because exercise can improve functional strength and balance, it can reduce the risk of falls and help to prevent fractures^21^. Lastly, moderate-to-high intensity exercise can increase muscle strength^34^. Notably, muscle strength is related to muscle mass, which is a strong determinant of bone size, volume, density and strength^35,36^. Therefore, if muscle strength is improved through exercise, the risk of fracture may be lowered.

The benefits of exercise likely outweigh its risks, and reports of fractures caused by exercise are rare. Joint pain, muscle pain, and sprains are the most common side effects associated with exercise^37,38^. In a previous study, there was only one report of a fall during exercise (in a patient with spinal fractures), which did not result in serious side effects. Additionally, a previous systematic review concluded that exercise is sufficiently effective to outweigh potential harm^37^. Our study also showed that the anti-fracture effect of PA was greater than the risk of exercise-related fractures. These findings will help clinicians encourage their patients to increase their levels of PA.

A notable strength of this study was that it performed repeated measures analysis to investigate the association between interval changes in PA and the risk of fracture in the general population, using a large population-based database. In this study, the incidence of fractures was based on medical claims records collected at the national level, which are considered to be more accurate than retrospective data. Moreover, the study included adjustments for potential confounding factors, such as current smoking, alcohol consumption, and fracture history; extensive subgroup analyses were also performed.

There are some clinically significant findings in the present study. Continuous regular PA was found to be related with a markedly reduced risk of fracture. In addition, the effect of continuous regular PA was observed to be greater in individuals older than 65 years, male, and individuals with dyslipidemia or previous fracture history.

However, this study had some limitations. First, our research may have included inaccuracies because it was based on self-reported questionnaire data, which may not accurately reflect actual levels of PA among the participants. Nevertheless, self-reported questionnaires approximate PA at the population level, and the effectiveness of the IPAQ-based self-reported PA questionnaire has been confirmed in several studies^39,40^. Secondly, there are no data in the K-NHIS database that enabled evaluation of patients’ BMD. Third, because the study was based on real-world data, causes of interval changes in PA could not be assessed. Fourth, we did not include individuals within long-term care and/or assisted living. Fifth, we only analyzed the risk of fractures according to changes in regular PA. We did not consider time spent in PA, such as in sedentary behavior, light PA, moderate PA and vigorous PA. Also, in our study, as regular PA included both moderate and vigorous intensity PA together, the fracture risk could not be analyzed per each intensity of PA. Finally, caution is needed when generalizing these results to countries other than South Korea, and multi-ethnic cohort studies are warranted in the future.

## Conclusion

Continuous regular PA is associated with fracture prevention. Specifically, it is more effective in men, individuals ≥ 65 years, and those with previous fractures and dyslipidemia.

## Data Availability

Data are available from the Korea National Health Insurance Sharing Service Institutional Data Access/Ethics Committee (https://nhiss.nhis.or.kr/bd/ay/bdaya001iv.do) for researchers who meet the criteria for access to confidential data. However, in accordance with Korean law, the study authors are not permitted to transfer any data files to a third party.
